# Assessment of the Phenylketonuria (PKU)-Associated Mutation p.R155H Biochemical Manifestations by Mass Spectrometry-Based Blood Metabolite Profiling

**DOI:** 10.32607/20758251-2019-11-2-42-46

**Published:** 2019

**Authors:** O. A. Baturina, A. A. Chernonosov, V. V. Koval, I. V. Morozov

**Affiliations:** Joint Center for genomic, proteomic and metabolomics studies, Institute of Chemical Biology and Fundamental Medicine SB RAS, Lavrentiev Ave. 8, Novosibirsk, 630090, Russia; Department of Natural Sciences, Novosibirsk State University, Pirogova Str. 2, Novosibirsk, 630090 , Russia

**Keywords:** phenylketonuria, hyperphenylalaninemia, p.R155H, blood phenylalanine, blood carnitine, mass spectrometry, missense mutation

## Abstract

Homozygous siblings with different treatment histories represent an excellent model to study both the phenotypic manifestation of mutations and the efficacy of therapy. We compared phenylketonuria (PKU) manifestations in two different gender siblings who were homozygous carriers of a rare phenylalanine hydroxylase (*PAH*) mutation, p.R155H, subjected to different treatments. PKU caused by mild mutations may be easily underdiagnosed if the diagnosis is based solely on the phenylalanine (Phe) blood concentration. One of the described patients is an example of this diagnostic error. For reducing diagnostic errors, we suggest the use of more elaborate methods in screening practice, in particular mass spectrometric analysis of blood metabolites, the efficiency of which is demonstrated in the present study.

## INTRODUCTION

Type I phenylketonuria (classic) (PKU; MIM 261600) is an autosomal recessively inherited disease caused by a deficiency of phenylalanine hydroxylase (PAH; [EC 1.14.16.1]) activity [1, 2]. The deficiency of enzymatic activity is usually caused by mutations in the phenylalanine hydroxylase gene [1]. Incomplete conversion of phenylalanine to tyrosine due to deficient activity of PAH results in accumulation of phenylalanine and toxic products of alternative metabolic pathways, such as phenylpyruvate, vinyl acetate, phenyllactate, and phenylacetylglutamine, in tissues and biological fluids, which leads to the disease symptoms: dementia in particular. Common features of phenylketonuria also include a decreased concentration of tyrosine and a changed balance of amino acids in biological fluids. A widely used classification of PKU severity, which is based on the blood phenylalanine concentration, was proposed by C.R. Scriver and S. Kaufman [3]. According to N. Blau et al. [4], PKU is classified, depending on the blood phenylalanine concentration before the beginning of treatment, as a classic (severe) form at concentrations above 1,200 μmol, a moderate form at concentrations in a range of 900–1,200 μmol, a mild form at concentrations of 600–900 μmol, and hyperphenylalaninemia at concentrations of less than 600 μmol.

Neonatal biochemical screening is currently the most widely used procedure for early diagnosis of genetic diseases and prevention of their effects. The World Health Organization defined the social and economic prerequisites for including diseases in national neonatal screening programs [5]. Currently, neonatal screening for PKU, as one of the most common genetic diseases (rate of 1 : 10,000 to 1 : 25,000 among Caucasians [6]), is performed in most countries. In the Russian Federation, this screening involves several measurements of the blood plasma phenylalanine level, the first of which is carried out on day 4 or 5 after birth. If an elevated level is detected, the measurement is repeated. However, the blood phenylalanine-based diagnostics may be unreliable, in particular at borderline levels. The accurate diagnosis requires more advanced methods, especially in the case of mutations with a relatively high residual PAH activity when the blood phenylalanine level increases slightly, remaining less than 500 μmol, which is not enough for an unambiguous diagnosis. Mass spectrometric analysis of blood metabolites can provide a more accurate diagnosis based on assessing the concentrations of not only phenylalanine, but also tyrosine and other amino acids, which, in particular, provides the phenylalanine to tyrosine ratio. The diagnosis based on metabolite levels should be confirmed by genetic analysis with identification of *PAH *gene mutations and their inheritance patterns by determining the nucleotide sequences of the appropriate loci.

Currently, the main and most widely used method for PKU correction is diet therapy [7, 8] aimed primarily at preventing damage to the central nervous system (CNS), which leads to the degradation of mental abilities. To achieve this goal, diet therapy should begin no later than a few weeks after birth. Being generally quite effective, this diet restricts the intake of animal-derived proteins, which may lead to decreased concentrations of free carnitine (C0) and acylcarnitines (C2–C18) and, correspondingly, to impaired metabolism and mitochondrial dysfunction in some patients [9].

In this study, we determined blood metabolite levels in the presence and absence of diet therapy in two siblings who were homozygous carriers of a PKU-associated mutation, p.R155H, of the *PAH *gene, using mass spectrometry of dried blood samples. Related homozygous patients with different histories of diet therapy may be considered as a unique model for studying both the phenotypic manifestation of the mutations and the efficacy of therapy.

## EXPERIMENTAL

Both patients were children of the same parents. The girl (1), who was born in Russia in June 2009, was diagnosed with PKU upon neonatal biochemical screening; she received a diet with a limited content of phenylalanine from the second month of life. The boy (2), who was born in Uzbekistan in July 2001, was not diagnosed with PKU upon neonatal screening and did not receive diet therapy. Blood samples for mass spectrometric analysis were taken in 2010, approximately one year after the start of diet therapy for patient 1. Simultaneously, the physical development and CNS status of the patients were assessed, which did not reveal any serious abnormalities. The study was performed in accordance with the requirements of the Medical Ethics Committee of the Institute of Chemical Biology and Fundamental Medicine of the Siberian Branch of the Russian Academy of Sciences (ICBFM SB RAS).

Upon neonatal screening, blood samples were collected by paper filters (Perkin Elmer, Finland) and the blood phenylalanine concentration was determined using a Delfia/Victor counter and a reagent kit from the manufacturer (Wallac Oy, Finland). Genomic DNA was isolated from blood as previously described [10]. Mutations in the *PAH *gene were identified by both DNA strands Sanger sequencing of PCR amplification products from all exons and adjacent intron regions of the *PAH *gene using a BigDye Terminator v.3.1 Cycle Sequencing kit and an ABI 3130xl genetic analyzer (Applied Biosystems, USA) at the Genomics Core Facility of the ICBFM SB RAS. The nucleotide DNA sequences of both patients were identical and corresponded to the known *PAH *gene sequences, except for the p.R155H mutation. The parents’ DNA sequences confirmed inheritance of the p.R155H mutation. Mass spectrometric analysis of phenylalanine, tyrosine, and acylcarnitine levels was performed using a standard procedure [11] at the Mass Spectrometry Core Facility of the ICBFM SB RAS. Samples and internal standards were prepared using an Amino Acids and Acylcarnitines kit #55000 for newborn screening (Chromsystems Instruments & Chemicals, Germany); samples were prepared according to the kit manufacturer’s protocol. The analysis was performed using an Agilent 6410 QQQ tandem mass spectrometer equipped with an ESI ionization system, which was coupled to an Agilent 1200 liquid chromatography system (Agilent Technologies, USA). Quantitative analysis was carried out in the multiple reaction monitoring (MRM) mode, with a total analysis time of 2.5 min. The signal was acquired and analyzed using the MassHunter v.1.3 software.

## RESULTS AND DISCUSSION

The point mutation p.R155H is a substitution of G for A in exon 5 of the *PAH *gene, which results in the replacement of arginine with histidine in the PAH catalytic domain. According to the data from http:// www.biopku.org [12], the residual activity of the enzyme carrying the mutation remains rather high (approximately 44% of the initial activity). Therefore, the p.R155H mutation is considered to be associated with hyperphenylalaninemia with a relative genotype-phenotype correlation coefficient of 8 on the scale proposed by Guldberg et al. [1]. This rare mutation was previously described only in one case in compound with the p.D143G mutation in a hyperphenylalaninemia patient [13]. We studied two siblings who were homozygous carriers of the p.R155H mutation, one of whom was subjected to diet therapy, while the other was untreated. Being homozygous mutation carriers, these patients provide a unique opportunity to directly study the phenotypic manifestation of the mutation, while significant differences in therapy may be used to assess its comparative efficacy.

The initial phenotypic manifestation of the p.R155H mutation in patients 1 and 2 was somewhat different, despite the same *PAH *locus genotype and common pedigree. The blood phenylalanine concentration in patient 1 upon neonatal screening was 1,100 μmol; the patient was diagnosed with moderate PKU, and diet therapy was prescribed. Patient 2 was not diagnosed with PKU upon neonatal screening, which was performed in the Republic of Uzbekistan, possibly due to different timing and standards of screening. In 2010, the blood phenylalanine concentration in patient 2 was 497 ± 13 μmol (*Table 1*), which corresponds to severe hyperphenylalaninemia. Therefore, if only the blood phenylalanine level is used as a criterion, it may be concluded that the p.R155H/p.R155H genotype can manifest itself as a fairly wide range of symptoms, from mild hyperphenylalaninemia to moderate PKU, depending on the other patient’s biochemical features associated with gender, feeding in the neonatal period, etc. In this case, neonatal screening that is based on the blood phenylalanine level may miss the disease, as in the case of patient 2.

**Table:** Blood metabolite concentrations measured by mass spectrometry

Metabolite	Patient 1, μmol/L	Patient 2, μmol/L	**Median concentration in healthy children (5th–95th percentile), μmol/L** [14]
Phe	298 ± 10	497 ± 13	45 (32–64)
Tyr	43 ± 2	86 ± 2	84 (48–159)
Phe/Tyr	6.9 ± 0.3	5.8 ± 0.1	
Free carnitine (C0)	31.7 ± 0.4	44.0 ± 1.1	15.5 (9.2–26.4)
Acetylcarnitine (C2)	10.4 ± 0.7	11.9 ± 0.3	17.3 (10.1–29.4)
Propionylcarnitine (C3)	1.48 ± 0.04	2.68 ± 0.2	1.42 (.81–2.56)
Butyrylcarnitine (C4)	0.11 ± 0.02	0.15 ± 0.02	0.19 (0.12–0.30)
Isovalerylcarnitine (C5)	0.11 ± 0.01	0.11 ± 0.01	0.09 (0.06–0.17)
Hexanoylcarnitine (C6)	0.04 ± 0.01	0.09 ±0.002	0.05 (0.02–0.10)
Octanoylcarnitine (C8)	0.02 ± 0.002	0.06 ± 0.02	0.05 (0.03–0.08)
Decanoylcarnitine (C10)	0.02± 0.002	0.07 ±0.002	0.07 (0.04–0.13)
Dodecanoylcarnitine (C12)	0.03 ± 0.02	0.09 ±0.002	0.08 (0.04–0.19)
Tetradecanoylcarnitine (C14)	0.06 ± 0.003	0.07 ±0.002	0.18 (0.11–0.31)
Hexadecanoylcarnitine (C16)	0.56 ±0.02	0.53 ±0.02	2.93 (1.65–4.76)
Octadecanoylcarnitine (C18)	0.34 ±0.01	0.32 ± 0.01	0.86 (0.52–1.43)

Values are presented as mean ± SEM of 5 or 6 independent measurements.

The effective use of mass spectrometry for accurate diagnosis of PKU, in particular at intermediate blood phenylalanine concentrations, was described in studies by Chace et al. [15, 16]. Instead of the phenylalanine concentration, they suggested using the phenylalanine to tyrosine concentration ratio as a more accurate and selective diagnostic criterion, because the changes in overall amino acid levels are corrected in this case. The phenylalanine to tyrosine concentration ratio in the blood normally ranges from 0.49 to 0.93, rising to 1.3– 14.3 in PKU patients [15, 16]. We detected high values of this parameter, which clearly and unambiguously indicated PKU in both patient 1 (6.9 ± 0.3) and patient 2 (5.8 ± 0.1) (*Table 1*). At the same time, the patients’ parents (heterozygous carriers of mutations) showed the ratio typical of healthy people: 0.9 ± 0.1 (mother) and 0.7 ± 0.02 (father).

We also determined blood carnitine levels in the patients (*Table*) and compared them with the data of 20 healthy children [17]. As previously shown, carnitine levels may be a helpful criterion in the identification of congenital metabolic disorders and the levels vary significantly in different metabolic disorders [18]. It was also noted that carnitine concentrations significantly decrease in the blood of PKU patients with a phenylalanine-restricted diet not supplemented with carnitines [19]. In our patients, total carnitine levels were slightly decreased (44.9 μmol in patient 1 and 55.2 μmol in patient 2) compared to the normal levels of 60–100 μmol [20]. Total acylcarnitine levels (13.2 μmol in patient 1 and 13.9 μmol in patient 2) fully corresponded to the normal values. We determined potential carnitine deficiency using the ratio of acylcarnitine and free carnitine concentrations. The values (0.42 in patient 1 and 0.33 in patient 2) were significantly below the upper limit of the normal value (0.6), which indicates sufficient levels of free carnitine, as well as the normal proportion of acylcarnitines. Therefore, acylcarnitine concentrations in our homozygous carriers of p.R155H were within their normal range; however, several studies [21, 22] have demonstrated that acylcarnitine concentrations in patients with classic PKU are significantly different from normal values.

## CONCLUSION

We examined two homozygous siblings with the PKU-associated mutation p.R155H who had different treatment histories: one was subjected to diet therapy, and the other did not receive treatment. If the blood phenylalanine concentration is used as the only diagnostic criterion for PKU, the condition should be classified as hyperphenylalaninemia in one patient and as moderate PKU in the other. At the same time, the phenylalanine to tyrosine concentration ratio enabled a reliable diagnosis of PKU in both patients. Therefore, the blood phenylalanine level alone cannot be a reliable criterion for the diagnosis of PKU in all cases and should not be used as the sole diagnostic criterion. Our findings confirm the possibility of using the phenylalanine to tyrosine concentration ratio as a more accurate and reliable criterion for neonatal screening. We also demonstrated that mass spectrometric analysis may be used as the main method of neonatal screening for PKU.

The determined blood concentrations of free and acylcarnitines indicate that homozygous carriers of the p.R155H mutation probably do not experience significant limitations in energy metabolism in cells, in contrast to patients with classic or moderate PKU.

## Abbreviations

PKU: phenylketonuria
PAH: phenylalanine hydroxylase
CNS: central nervous system
C0: free carnitine
C2-C18: acylcarnitines
